# Chikungunya virus infections among travellers returning to Spain, 2008 to 2014

**DOI:** 10.2807/1560-7917.ES.2016.21.36.30336

**Published:** 2016-09-08

**Authors:** Maria Dolores Fernandez-Garcia, Mathieu Bangert, Fernando de Ory, Arantxa Potente, Lourdes Hernandez, Fatima Lasala, Laura Herrero, Francisca Molero, Anabel Negredo, Ana Vázquez, Teodora Minguito, Pilar Balfagón, Jesus de la Fuente, Sabino Puente, Eva Ramírez de Arellano, Mar Lago, Miguel Martinez, Joaquim Gascón, Francesca Norman, Rogelio Lopez-Velez, Elena Sulleiro, Diana Pou, Nuria Serre, Ricardo Fernández Roblas, Antonio Tenorio, Leticia Franco, Maria Paz Sanchez-Seco

**Affiliations:** 1 Centro Nacional de Microbiología, Instituto de Salud Carlos III, Madrid, Spain; 2 Current affiliation: Institut Pasteur de Dakar, Dakar, Senegal; 3 These authors contributed equally to this manuscript; 4 Current affiliation: World Health Organization, Geneva, Switzerland; 5 European Union Public Health Microbiology training programme (EUPHEM), European Centre for Disease Prevention and Control (ECDC), Stockholm, Sweden; 6 Hospital Carlos III-La Paz, Madrid, Spain; 7 ISGlobal, Barcelona Centre for International Health Research (CRESIB), Hospital Clínic-Universitat de Barcelona, Barcelona, Spain; 8 Department of Clinical Microbiology, Hospital Clínic, Barcelona, Spain; 9 Hospital Ramon y Cajal, Madrid, Spain; 10 Department of Microbiology, Hospital Universitari Vall d’Hebron, Barcelona, Spain; 11 Tropical Medicine and International Health Unit Drassanes-Vall d’Hebron, PROSICS Barcelona, Barcelona, Spain; 12 Fundación Jimenez Diaz, Madrid, Spain; 13 Current affiliation: Gorgas Memorial Institute, Panama City, Panama

**Keywords:** chikungunya, imported, Europe, dengue, Co-Infection, travel related

## Abstract

Since the first documented autochthonous transmission of chikungunya virus in the Caribbean island of Saint Martin in 2013, the infection has been reported within the Caribbean region as well as North, Central and South America. The risk of autochthonous transmission of chikungunya virus becoming established in Spain may be elevated due to the large numbers of travellers returning to Spain from countries affected by the 2013 epidemic in the Caribbean and South America, as well as the existence of the *Aedes albopictus* vector in certain parts of Spain. We retrospectively analysed the laboratory diagnostic database of the National Centre for Microbiology, Institute of Health Carlos III (CNM-ISCIII) from 2008 to 2014. During the study period, 264 confirmed cases, of 1,371 suspected cases, were diagnosed at the CNM-ISCIII. In 2014 alone, there were 234 confirmed cases. The highest number of confirmed cases were reported from the Dominican Republic (n = 136), Venezuela (n = 30) and Haiti (n = 11). Six cases were viraemic in areas of Spain where the vector is present. This report highlights the need for integrated active case and vector surveillance in Spain and other parts of Europe where chikungunya virus may be introduced by returning travellers.

## Introduction

Since the first outbreak in Tanzania in 1952, chikungunya has been endemic in some parts of Africa, south-east Asia and in the Indian subcontinent [1]. In 2013, however, the first documented autochthonous transmission of chikungunya virus was reported in the Caribbean island of Saint Martin [2] and since then the infection has spread quickly to other countries and territories of the Caribbean as well as to North, Central and South America [2]. As at 31 December 2014 (the final day of our study), 24,682 confirmed autochthonous cases and 1,118,763 suspected cases of chikungunya in the Americas had been reported by the Pan American Health Organization (PAHO) [3]. A large majority of these suspected autochthonous cases (n = 802,714; 72%) were reported from the Caribbean, in particular from the Dominican Republic (539,099; 67%).

A chikungunya outbreak in 2005 in Réunion, a French overseas department and region, affected 266,000 people (ca 35% of the population), including 783 cases imported to metropolitan France [4]. During this outbreak, a mutation (A226V in the chikungunya virus E protein) that improved the replication in *Aedes albopictus* was observed, giving rise to a new virus variant whose fitness in this mosquito was increased [5].

The transmission of chikungunya virus to humans occurs mainly through bite of infected *Ae. aegypti* or *Ae. albopictus* mosquitoes, which can also transmit dengue virus. In Europe, *Ae. albopictus* is established primarily around the Mediterranean basin [6,7] and has been demonstrated to be competent for chikungunya virus transmission in this region [8]. This has resulted in locally acquired infections in Italy (Emilia Romagna region, 2007) as well as in France (Var and Montpellier, 2010 and 2014 respectively) [9-11].

In Spain, despite the presence of *Ae. albopictus* in the eastern Mediterranean regions (Catalonia, Valencia, Murcia, Balearic Islands) [12] and recently in the southern region of Andalusia [13] and in the Basque Country [14], no autochthonous transmission of chikungunya virus has been reported. However, the risk of autochthonous transmission of chikungunya establishing in Spain may be elevated due to the large numbers of travellers returning to Spain from countries affected by the 2013–14 epidemic in the Caribbean and South America [2], as well as the existence of the competent vector in certain parts of Spain. In order to further assess this risk and better understand the epidemiological and laboratory characteristics of imported chikungunya cases, we retrospectively analysed the laboratory diagnostic database of the National Centre for Microbiology, Institute of Health Carlos III (CNM-ISCIII) from 2008 to 2014.

## Methods

### Case definition

The case definition used followed the guidelines provided by the Spanish Ministry of Health [15] and the European Centre for Disease Prevention and Control (ECDC) [16]. 

A suspected case was a patient meeting clinical criteria (acute onset of fever (> 38 °C) and severe arthralgia not explained by other medical conditions) and epidemiological criteria (residing in or having visited epidemic or endemic areas). 

A confirmed case was a patient meeting the laboratory criteria, irrespective of the clinical presentation (with at least one of the following: virus isolation, presence of viral RNA, presence of virus-specific IgM antibodies in a single serum sample collected in acute or convalescent stage, or fourfold rise in IgG titres in samples collected at least 15 days apart) [15]. Patients with chikungunya virus or chikungunya viral RNA detected in serum were considered viraemic.

### Serology and molecular analysis

The presence of IgM or IgG antibodies against chikungunya virus was detected by indirect immunofluorescence (Euroimmun, Germany).

The presence of dengue virus IgG and IgM antibodies was detected using IgM capture ELISA and IgG indirect ELISA tests [17].

The presence of chikungunya viral RNA was detected by an in-house real-time reverse transcription-PCR (RT-PCR) and confirmed by two established PCR protocols [18].

### Dengue diagnostics

Dengue viral RNA was detected using RT-PCR [19]. Dengue nonstructural protein 1 antigen (NS1) was detected by enzyme immunoassay (Platelia Dengue NS1 Ag, Biorad).

### Data used

All data from samples received for diagnosis and surveillance of imported viral infections in the CNM-ISCIII between 1 January 2008 and 31 December 2014 were included in the study. Samples and data were codified with a unique ID to ensure the anonymity of patients.

We accessed the CNM-ISCIII database, in which information was typically collected on sex, age, date of onset of symptoms, date of specimen collection, travel destination and hospitalisation. We also contacted local health authorities and hospitals to retrieve information on travel destination. Samples included acute and convalescent sera. We used international travel statistics obtained from a 2014 World Tourism Organization report [20] to estimate incidence rates of chikungunya virus-infected travellers returning to Spain.

## Results

### Infections reported and patient characteristics

During the study period (1 January 2008 to 31 December 2014), a total of 1,371 suspected chikungunya cases were identified (179 in 2008, 134 in 2009, 177 in 2010, 129 in 2011, 93 in 2012, 87 in 2013 and 572 in 2014). The most frequently reported reasons for travel were work (aid workers, missionaries, others), visiting friends and relatives and tourism. In 2008–13, the median number of suspected cases was 131 (range: 87–179). Of the 1,371 suspected cases in 2008–14, 42% (n = 572) were reported in 2014 alone.

**Figure 1:**
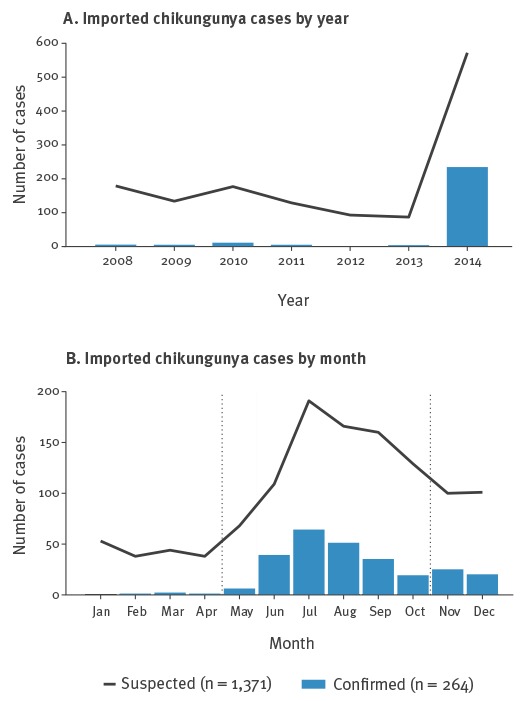
Number of suspected and confirmed imported chikungunya cases by year and by month, Spain, 2008–14 (suspected n = 1,371; confirmed n = 264)

A total of 264 (19%) suspected cases were laboratory confirmed by CNM-ISCIII during the study period. Of the confirmed cases, the majority were female (n = 159; 60%), with a median age of 43 years (range: 1–93). The number of suspected and confirmed imported cases from 2008 to 2014 in Spain is shown in Figure 1A. During 2008–13, 30 imported chikungunya cases were laboratory confirmed (6 in 2008, 4 in 2009, 11 in 2010, 5 in 2011, 1 in 2012, 3 in 2013) with a median of 5 (range: 1–11) cases identified per year, while in 2014 there were 234 confirmed imported cases. The ratio of confirmed to suspected cases shifted from 1:28 in 2008–2013 to 1:3 in 2014. The distribution of imported cases by month during our study period in Spain shows that the number of confirmed and suspected cases reached its peak during July (Figure 1B).

In panel B, the two vertical lines indicate the start and end of the active period for the chikungunya virus vector *Aedes Albopictus* in Spain (May to October).

### Travel history

The travel history of the patients is detailed in Table 1. Between 2008 and 2013, the travel destination was known for 19 of the 30 confirmed cases: 17 cases had travelled to Asia (Indonesia (n = 5), India (n = 5), Myanmar (n = 2), Thailand (n = 1), Philippines, (n = 1), other Asian countries not specified (n = 3)) and two cases had travelled to Africa (Cameroon and Equatorial Guinea). Of the 234 cases in 2014, the travel history of 220 patients (94%) was known: 154 cases (70%) reported having visited the Caribbean during the incubation period [16], 59 cases had visited Central and South America (Venezuela, Colombia, Mexico, Nicaragua, Peru, El Salvador, Panama), five had visited Africa (Angola, Madagascar, Equatorial Guinea, and Mozambique) and two had visited Asia (India, unspecified). No autochthonous infections acquired in Spain were identified or reported by the health authorities.

**Table 1:** Number of confirmed chikungunya cases (n = 264) and incidence rate of chikungunya among travellers returning to Spain, by travel destination, 2008–2014

Travel destinations – countries and territories	Mean annual number of travellers from Spain during 2008–13^a^	2008–13		2014	
		Number of cases	Incidence rate per 100,000 travellers arriving in Spain	Number of cases	Incidence rate per 100,000 travellers arriving in Spain
Americas					
Venezuela	610,333	0	0	30	4.92
Haiti	317,666	0	0	11	3.46
Dominican Republic	4,421,000	0	0	136	3.08
Guadeloupe	405,000	0	0	2	0.49
Martinique	487,000	0	0	1	0.21
Colombia	2,200,000	0	0	3	0.14
Dominica	77,250	0	0	1	1.29
Puerto Rico	3,125,750	0	0	3	0.10
Nicaragua	1,083,000	0	0	1	0.09
El Salvador	1,196,000	0	0	1	0.08
Panama	1,515,250	0	0	1	0.07
Peru	2,581,000	0	0	1	0.04
Americas (unspecified, but excluded North America)	57,849,000	0	0	21	0.04
Mexico	23,365,000	0	0	1	0.00
Africa					
Mozambique	1,911,000	0	0	1	0.05
Madagascar	218,250	0	0	1	0.46
Angola	478,000	0	0	2	0.42
Cameroon	664,666	1	0.15	0	0
Equatorial Guinea^b^	No data	1	NC	1	NC
Asia					
India	6,377,750	5	0.07	1	0.02
Asia (unspecified)	235,587,000	3	0.00	1	0
Indonesia	7,874,750	5	0.06	0	0
Myanmar	54,875	2	0.36	0	0
Thailand	21,016,750	1	0.00	0	0
Philippines	4,097,750	1	0.02	0	0
No travel destination specified					
Cases with missing data	NA	11	NA	14	NA
**Total number of cases**	NA	**30**	NA	**234**	NA

NA: not applicable; NC: not calculated, as no denominator data.

^a^ Source: [20] for data from 2008 to 2012 and [45] for data from 2009 to 2013. At the time of analysis, traveller data were not available for 2014.

^b^ Traveller data were not available for Equatorial Guinea for 2008–13.

The incidence rates of chikungunya in travellers worldwide returning to Spain are shown in Table 1. The highest incidence rate in 2014 was seen in travellers returning from Venezuela, with 4.92 cases per 100,000 travellers, followed by those who had travelled to Haiti (3.46/100,000) and the Dominican Republic (3.08/100,000). In contrast, during 2008–13, the highest incidence rate was in travellers returning from Myanmar, with 0.36 cases per 100,000 travellers.

Information about reason for travel was available for a limited number of suspected cases in 2014 (n = 66). Among those 66 suspected cases, the most frequently reported reasons for travel were visiting friends and relatives (n = 27), tourism (n = 25) and work (n = 14; aid workers, missionaries, others).

### Region of notification and presence of vector

**Figure 2:**
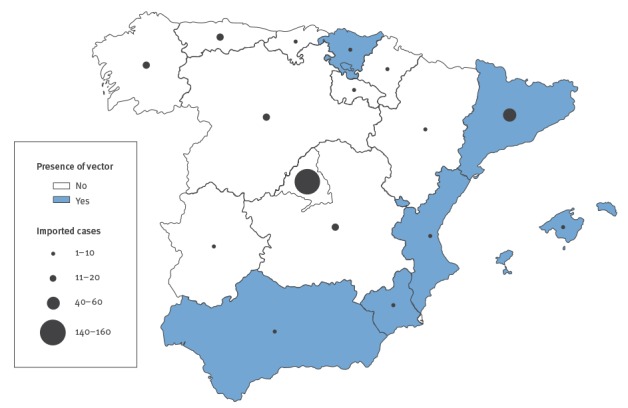
Geographical spread of confirmed imported chikungunya cases (n = 264) and presence of chikungunya virus vector *Aedes albopictus* in autonomous regions of Spain, 2008–14

Overall, 120 hospitals in all Spanish regions referred samples to the CNM-ISCIII during the study period. Of the 264 cases confirmed between 2008 and 2014, Madrid and Catalonia had the largest number of notifications (150 (57%) and 44 (17%), respectively). A total of 66 of the cases (25%) were reported in regions where *Ae. albopictus* is present (Catalonia, Autonomous Community of Valencia, Basque Country, Murcia, Balearic Islands, and since 2013 southern Andalusia) (Figure 2). Of these 66 cases, six were viraemic (PCR positive) when presenting to clinicians in Spain. 

In 2014, during the active period of *Ae. albopictus* in Spain (May–October), we confirmed a total of 33 imported cases in regions with vector presence, three of whom were viraemic when presenting to clinicians in Spain. 

### Serological diagnostics

Of the 33 confirmed imported cases in regions in which *Ae. albopictus* was present, 30 had anti-chikungunya virus IgM antibodies, suggesting recent infection.

Serology was performed on 1,147 of the 1,371 (84%) suspected imported cases. Of the 1,147 tested, 235 (20%) were positive for IgM. Of the 235 IgM-positive samples, 11 (5%) also tested positive by the molecular methods described. 

From 36 IgM-positive imported cases with known date of symptoms onset, samples were collected within 0 to 37 days after onset (median: 7 days).

Paired serum samples were available for 37 confirmed cases, where the time elapsed between the first and second samples ranged from 2 to 210 days. The results of the IgM and IgG assays are shown in Table 2. The time taken for an initially positive IgM test to become negative ranged from 93 to 181 days in our study. Among cases for whom the first sample was IgG positive, some remained IgG positive for up to 210 days, when the second sample was taken. Of 37 paired serum samples, 15 demonstrated seroconversion within 14 to 60 days.

**Table 2:** Indirect immunofluorescence results and interval between the dates the first and second samples were taken, for convalescent paired samples from 37 confirmed cases, Spain, 2008–14

Indirect immunofluorescence results		Number of samples	Mean time in days^a^ (range) between the dates the first and second samples were taken, post symptom onset
First sample	Second sample		
IgM			
Pos	Pos	17	43 (7–79)
Neg	Pos	4	22.5 (16–29)
Neg	Neg	4	117 (53–181
IgG			
Pos	Pos	17	108 (6–210)
Neg	Pos	15	38 (16–60)
Pos	Neg	1	58^b^ (NA)

NA: not applicable; Neg: negative; Pos: positive.

^a^ Unless otherwise specified.

^b^ The second sample was taken 58 days after symptom onset. The date the first sample was taken was not available.

### Molecular diagnostics

Molecular diagnosis was carried out for samples from 481 (35%) of the 1,371 suspected cases: viral genome was detected by RT-PCR in 39 (8%) of the 481 tested. Of the 39 patients with a chikungunya virus-positive PCR, 11 had a known date of symptom. The samples were collected 0–3 days after symptom onset except for one, which was collected after six days. 

Of the 235 patients whose infection was confirmed by IgM, 86 were negative by PCR. For these 86 patients, the median time from onset of symptoms to sample collection was four days (range: 0–35).

### Concurrent infection

Of the 1,371 suspected chikungunya cases reported during the study period, 817 were also tested for the presence of anti-dengue virus IgG and IgM antibodies, dengue RNA and/or dengue nonstructural protein 1 antigen (NS1). In 2014, 41% (234/572) of the suspected chikungunya cases were tested for dengue virus infection while in the rest of the study period (2008–13), 73% (583/799) of the suspected chikungunya cases were tested for dengue virus infection. Dengue RNA, NS1 and/or anti-dengue virus IgM antibodies were found in 116 of the 1,371 suspected chikungunya cases. During 2008–13, a total of 87 imported dengue cases were laboratory confirmed (9 in 2008, 13 in 2009, 28 in 2010, 9 in 2011, 12 in 2012, 16 in 2013); in 2014, there were 29 confirmed imported dengue cases. The distribution of dengue imported cases by month in 2014 showed that the number of confirmed cases reached its peak during August (data not shown). A total of 13/29 confirmed dengue cases in August 2014 reported having visited the Americas. 

Of the 116 dengue cases, five were also confirmed as positive for chikungunya virus, one in 2010 and four in 2014. Of these five, three had anti-dengue virus and anti-chikungunya virus IgM, one had anti-dengue virus IgM and chikungunya viral RNA, and one had dengue viral RNA and chikungunya viral RNA. The coinfected patients in 2014 had returned from Venezuela (n = 2) and the Dominican Republic (n = 2). The patient coinfected in 2010 had returned from the Philippines (Table 3).

**Table 3:** Dengue virus and chikungunya virus laboratory results from coinfected confirmed imported cases, Spain, 2008–14 (n = 5)

Case	Confirmed dengue	Confirmed chikungunya			Travel destination	Year
		IgM	IgM plus PCR	PCR		
1	IgM positive	Pos	Neg	Neg	Venezuela	2014
2		Pos	Neg	Neg	Philippines	2010
3		Pos	Neg	Neg	Dominican Republic	2014
4		Neg	Neg	Pos	Dominican Republic	2014
5	IgM and PCR positive	Neg	Neg	Pos	Venezuela	2014

Neg: negative; Pos: positive.

## Discussion

We have described a 7.8 fold increase in the number of imported chikungunya virus infections from 2008–13 (30 cases) to 2014 (234 cases) in Spain, with the number in 2014 being the highest recorded in the country. Every year since 2006, imported chikungunya cases have been identified among travellers returning to Spain. During 2006 to 2007, 29 laboratory-confirmed imported cases were diagnosed among a cohort of 308 travellers with symptoms compatible with acute or recent chikungunya virus infection on their return to Spain [21]. The majority of these cases (n = 20) had visited India or the Indian Ocean Islands. Similarly, during 2008 to 2013, most imported cases (17/19) arrived from Asia. As a result of an ongoing outbreak in the Americas, with more than 780,000 suspected cases by 31 October 2014 [22], however, the majority of cases imported to Spain in 2014 were from the Caribbean and northern parts of South America [23], demonstrating the potential of this large chikungunya outbreak to affect Spain.

The Caribbean is a popular travel destination for Spanish travellers during the spring and summer months. It is also a destination for migrants living in Spain, who travel back home to visit friends and relatives, as shown by the large proportion of chikungunya cases in our study who had visited friends and relatives (27/66). Spain has a dynamic population of Latin American and Caribbean immigrants with permanent residency in Spain who frequently travel to their country of origin. Immigrants from the Caribbean, especially the Dominican Republic, have represented the one of the largest proportion of immigrants in Spain in recent decades and a continued growth of Dominican immigration is predicted for the future [24]. This correlates with the high number of confirmed chikungunya cases from the Dominican Republic (136/264) seen in our study. Furthermore, migrants visiting friends and relatives are less likely than tourists to seek travel health advice, and therefore to take preventive measures during their stay, representing an important gateway for the entry of chikungunya virus in Europe [25]. This challenge requires appropriate countermeasures such as targeted guidance to these groups of travellers at risk and early screening.

During 2015, however, the virus spread from the Caribbean to South and Central America, and more countries have become affected. The virus spread in Colombia, Venezuela, Brazil and other South American countries during the summer of 2015. In Brazil, where in 2014 a total of 3,657 cases distributed in eight municipalities were reported, in 2015 the number of cases increased more than fivefold, to 20,661 cases in 84 municipalities, mainly in north-east and south-west states [26].

Appropriate surveillance and investigation of imported chikungunya cases could also aid in understanding better the epidemiological and virological dynamics of the outbreak in the Caribbean and Latin American countries. Returning travellers can serve as indirect sentinels to monitor the geographical spread of the outbreak in the Americas. For example, in our study 52% of imported cases came from the Dominican Republic, in line with PAHO´s report in October 2014 that showed 62% of cases in the Americas stem from this country [22]. Similarly, in late 2014, 13% of chikungunya virus-infected travellers were returning from Venezuela, around the time the country was facing a large re-emergence of vector-borne diseases [27]. If we look in 2015 for chikungunya cases in Spain imported from Venezuela, the percentage would probably be larger due to the large epidemic there. 

Sharing of data gained through analysis of returning travellers could support countries where no or scarce data on chikungunya have been reported. In our study, for example, we saw an imported case from Mozambique, a country that only recently demonstrated circulation of chikungunya virus [28]. We also saw cases in travellers returning from Colombia, El Salvador and Nicaragua, which have reported local transmission, and from Panama, which had reported imported cases in 2014 [3,29]. While this can serve as an indication, using travel information alone is not sufficient to definitively determine the country source of infection as travel is seasonal to most destinations and the epidemic may have been introduced before infected travellers returned.

Although the chikungunya outbreak in the Caribbean has led to an increase in the number of cases in Spain, enhanced surveillance may have also contributed to the rise. Chikungunya was classified a notifiable disease in 2013 by the Consejo Interterritorial del Sistema Nacional de Salud and became law in 2015, after our study period [30,31]. This may have led to greater awareness among patients and physicians, resulting in better ascertainment of suspected cases. Before the outbreak in the Caribbean, 1 in 28 suspected chikungunya cases were confirmed positive in the ISCIII whereas in 2014, the ratio was 1 in 3 (confirmed vs suspected cases). In 2008–13, of the 799 cases notified only 30 were confirmed, whereas in 2014, 234 of 572 notified cases were confirmed. Similarly, in south-eastern France, an increase in the number of suspected cases was noted in 2010 due to enhanced surveillance for chikungunya implemented after autochthonous chikungunya transmission was reported [10].

Although autochthonous transmission of chikungunya has been documented in south-eastern France in 2010 and 2014 [8,10] as well as in Italy in 2007 [9], Spain has not reported local transmission of chikungunya virus, despite the presence of *Ae. albopictus* mosquitoes in the country [32]. Chikungunya virus strains with the A226V viral mutation have not yet been described in America, as the circulating genotype is the Asian one and not the East/Central/South African (ECSA) genotype, in which the mutation arose [5]. This mutation should be considered in risk assessments of local transmission in Spain. Our data show that most travellers returned to Spain during the warm months of June to September, coinciding with the period of activity for *Ae. albopictus* (May–October). In 2014, the highest number of confirmed imported cases of chikungunya were in areas in which the vector was present (n = 66; Catalonia, Autonomous Community of Valencia, Murcia, Basque Country, Balearic Islands and Andalusia). Importantly, six of these 66 confirmed cases were positive by PCR. Such viraemic travellers are potential disseminators of the virus through bites of vectors, or by donating blood [33]. The ability of infected vectors to effectively overwinter until the next hot season further adds to the importance of imported chikungunya [34]. Considering that autochthonous transmission in France in 2010 occurred following importation of only two cases of chikungunya, the high number of infected travellers returning to Spain highlights the increased risk of autochthonous transmission becoming established. It is important to note that, to date, countries affected by localised outbreaks in the EU have been able to contain further spread of the disease [10,35].

Serological diagnosis can be performed by detection of specific IgM antibodies in serum from four to five days after the onset of symptoms, or a fourfold rise in chikungunya- specific IgG antibody titre in a paired serum sample. Chikungunya-specific IgM can persist for months, in particular in patients with persistent arthralgia [36]. The reported sensitivity of the commercial Immunofluorescence test used for detection of chikungunya-specific IgM has been 98.3%, with a sensitivity of 96.9% [37]. The specificity and sensitivity for the detection of IgG is 100.0% and 95.4%, respectively [37]. In our study, we observed that IgM can be detected from a median of seven days after onset of symptoms and that IgG can be detected from 16 days after the first sample is taken. This highlights that if specimens are collected very early in the course of the illness and tested only for IgM antibodies, serological diagnostic testing may not detect cases. In convalescent samples, we observed that IgM can persist up to 79 days. This should be taken into consideration when sampling and testing for chikungunya virus in returning travellers. Further study is also merited as complete case information for this analysis was available only for a limited subset of cases.

As chikungunya virus infection has similar symptoms as dengue and both viruses can circulate in the same area, chikungunya fever has often been mistaken for dengue. Confirmed dengue imported cases among travellers seen in our study support the suspicion of dual endemic circulation of dengue and chikungunya viruses in the Caribbean. Coinfections have been reported in Saint Martin, where 2.8% of chikungunya cases were reported as dengue coinfections in December 2013 to January 2014 [38]. In 2010, south-eastern France reported the concomitant emergence of dengue and chikungunya viruses, with two autochthonous infections reported for each [10,35]. Here we report five coinfections, four occurring in 2014 and one in 2010. Coinfections in travellers returning to Europe from the Indian Ocean Region in 2006 have been reported [39]. In addition, imported cases of Mayaro virus infection, an American alphavirus, were described in Germany in 2013 [40], France in 2010 [41] and the Netherlands in 2008 [42]. An imported case of O’nyong nyong virus infection, an African alphavirus, was described in Germany in 2013 [43]. Differential diagnosis of alphaviruses is therefore important and physicians should familiarise themselves with their clinical presentation.

The importance of coinfection is further highlighted as *Ae. albopictus* is also a recognised vector with competence and capacity to transmit dengue virus. With the increasing spread of *Ae. albopictus* in southern Europe, the risk of the establishment of these two arboviruses in Europe has also increased [44]. Continued awareness of other emerging diseases is needed to ensure rapid detection and control. The implementation of a strategic national surveillance system adapted to the early detection of both chikungunya and dengue, combined with vector monitoring systems, is urgently needed.

Considering the intense international traffic between Spain and countries affected by the 2013–14 chikungunya outbreak, as well as immigration and the distribution of competent vectors, chikungunya virus is becoming a threat to Spain and neighbouring countries. This report highlights the need for integrated active case and vector surveillance in Spain and other parts of Europe where the virus may be introduced by returning travellers. We furthermore highlight our experiences with diagnosing samples from returning travellers and how they can be used to indirectly monitor the spread of the outbreak in the Americas. An outbreak of chikungunya in Spain could have a considerable impact on public health, the safety of blood donation supplies and on tourism. With the 2013–14 outbreak in the Americas, the number of chikungunya cases among travellers returning to Spain from affected areas is likely to continue to increase, especially in the summer season in Spain. Mediterranean countries should strengthen preparedness for the re-emergence and/or reintroduction of chikungunya virus and other *Aedes*-transmitted diseases, especially in regions where *Ae. albopictus* is present or could become established.
